# Account of Ann Fooks's Case of Ischuria and Vomiting of Urine

**Published:** 1814-02

**Authors:** 


*For the Medical and Physical Journal.*

### *Account of Ann Fooks's Case of Ischuria and Vomiting of Urine, by* Dr. Yeats; *concluded from p. 37.*

BETWEEN* the 6th of December and the 12th of April (periods mentioned in the case), at what precise time I do not recollect, a phial, capable of holding about six or eight ounces, was brought to me, filled with a fluid which it was told to me the girl had vomited, and which possessed all the sensible qualities of healthy urine in taste, color, and consistence. It was of a deeper color than straw, not perfectly transparent; and on standing, a cloud was observable in it. The vomiting of clear urine, such as that now described, should not be received as an objection for totally rejecting the account.† This girl had very frequently been, and at this particular period was, in a state of retching, by which the throat, gullet, and stomach, the superior part especially, (for retching did not always amount to full vomiting,) were pretty well at times denuded of their mucus, and the glands‡ exhausted of their secretions: it is not, therefore, at all impossible, that at times the urine was vomited before the secretion of mucus had again taken place in sufficient quantity to be detected in the vomited urine by ocular examination only. Moreover, the constantly varying morbid actions with which her constitution was harassed, and by which her different secretions were sometimes considerably diminished, and at others considerably increased, would undoubtedly have the same effect upon the mucous

* How often I visited the girl after the 6th of December, I cannot say, though I am now sure oftener than I had at first thought; but I did not see her after Mr. Gibbon's visit on the 19th of March, till after the urine had returned to its natural channel.

† The experiments of Mr. Home on the functions of the stomach, by which he discovered that that viscus contracts in the centre into two portions, give the idea that the stomach would at such times not discharge its contents in a mixed state, the more solid parts remaining at the bottom, while the more liquid would be vomited by the partial contraction of the upper portion. Any liquid then may be vomited comparatively clear in this way, as is seen in the phenomena of common vomiting. In this way it was, as related of the hysteric nun, that the urine was vomited unmixed with the food, although the vomiting occurred sometimes soon after her meals.—See paper on Ischuria, p. 364. See p. 31 of the case for Mr. Home's experiments.

‡ These glands are as likely to suffer a temporary torpor, by which there will be a suspension of their secretions, as well as the kidneys, liver, or any other gland in the body.

membrane membrane of the gullet and stomach: hence at times she brought up considerable quantities of mucus, and at others very little; and at all times the quantity of mucus thrown up by any person from the stomach will depend upon the degree of force with which its contents are rejected. The thing is, therefore, not inexplicable by any means; and in Dr. Senter's and Dr. Girdlestone's cases the urine vomited was found to be natural in every respect. Facts are of more avail than any theory. Here the urine was vomited as natural urine, and if in one or two cases, why not in a third? If vomiting of urine is impossible, the girl must be an impostor, and no other charge is necessary.

From about six weeks to the period of the return of the urine to its natural channel, the girl had taken many emetics under the direction of Mr. Palgrave, who of his own accord came to see her, no person directing any medicine for her at the time. At first, I have been since informed, the tartarised antimony was given, which proved too irritating and painful, and the ipecacuanha was substituted. The girl told me that she felt relief from their operation ultimately, by removing a great heaviness, weight, and oppression at the stomach; but their immediate effects were always painful. With the exhibition of the emetics I had nothing to do, beyond having said, "If you find relief from them, take them," any more than with a decoction of juniper berries, gum arabic, and barley, a cupful of which she took occasionally. The emetics, taken generally every other day, were commenced six weeks previous to the termination of the urinous vomiting; she having been subject to the vomitings from the commencement of the suppression up to that time, without taking any emetic.

The great distension of the stomach in this girl was evidently owing to a collection of great morbid effusions into it, and to a disengagement of air, giving the appearance of the meteorismus ventriculi. It is a fact well established, that the stomach shall remain in a distended state for weeks, and yet the food shall pass in regular course through its orifices. Food of difficult digestion, or matters effused into it by disease, shall lie in the stomach and oppress its functions for days; yet food more easily digested shall have undergone the actual changes, and have past through its lower orifice into the intestines for the nourishment of the body. The liver being sufficiently healthy to secrete bile of a proper color, the fæces would partake of the color, although the stomach shall be much diseased, provided the morbid secretions into the stomach did not pass down at the same time, and this would not always be simultaneous; and that the stomach

 possesses possesses the power of passing down one portion of its contents and retaining another, is well known. It must be remembered that this girl's stomach had been for a very long period in a diseased condition, from mechanical violence, yet possessing sufficient power to digest and transmit easily digested food, so that the body would be nourished, but by no means to such an extent as before the accident, when she had a healthier complexion, and a plumper appearance.*

Upon this morbid condition of the stomach supervened a deposition of urine into its cavity, in consequence of the suppression in October, from which time it was thrown up spontaneously under various appearances for many weeks, when, from the powers of the stomach becoming deficient to contract sufficiently to produce full vomiting, a greater accumulation of course took place, causing distension and oppression almost to suffocation, as she expressed it. Hence the obvious idea of an emetic occurred, the stimulus of which was necessary fully and more speedily to unload the stomach of its contents, and which, from the relief obtained, the girl requested might be repeated; and that the stomach does lose the power at times of acting sufficiently so as to produce full vomiting without the aid of an emetic, although loaded and oppressed, is a fact which requires no illustration, but which state would be attended, as in this indigent sufferer, with retching and nausea, until relief be obtained by full vomiting. A fit of indigestion from a gorged stomach has produced this state, when the cause being removed by an emetic, the effect ceases; but in this girl the cause was constantly recurring from the morbid condition of the stomach, and the diseased effusions into it.

That vomiting of urine has often occurred, I have no doubt. I have already stated what my ideas are on the subject: an altered state, however, of glandular secretions, particularly of those of the stomach, has some strong facts on its side. Physiologists know that the nature of the fluids of the stomach, and their chemical properties, are changed by an alteration in the action of its coats. The following is the statement the girl has given to me on this subject.

†Before taking any emetics, she used to vomit the urine clearer at some times than at others, two or three hours after

---

* Now above twelve years ago. I am informed that this was her appearance at that time.

† This account was taken from the girl at two different times, and before a different gentleman at each time, one a professional man, with the same result.

a slimy a slimy vomiting. That after having commenced the emetics, their immediate operation was followed by vomiting up a slimy urinous stuff, sometimes more, sometimes less in quantity, and sometimes of a greater and sometimes of a less urinous taste and smell. That the time which elapsed between this slimy vomiting, and vomiting of urine, was generally two hours. That between the times of taking the emetics she always vomited urine. That the urine which Mr. Gibbon saw had been thrown up from the stomach two hours and a half after the emetic had brought up the slimy stuff. She took no medicine from the time I saw her in December up to the time of commencing the emetics, except some opening pills which the druggist sent her. When the urine was brought up unmixed with the contents of the stomach, it was in quantity from six to ten ounces. When mixed with the various matters of the stomach, the whole mass thrown up would be very considerable.

Notwithstanding, then, all that has been said about the girl vomiting urine with an emetic, it does not appear that she vomited unmixed urine under the immediate operation of an emetic. She uniformly, when the emetic was taken, brought up the various matters of her stomach similar to what has been already stated; and in consequence of the emetic tasting, as she says, of something like peppermint, she did not perceive the urinous taste so strong in the slimy vomiting, as she used to do before they were commenced. The emetics were given from the excessive load and weight she felt in her stomach from the accumulation of morbid effusions. Their action brought up the mixed mass in one or two vomitings, and some time after this the vomiting would again take place, when the unmixed urine came up in a similar way, as it did before she took the emetics at all.* There was then no difference whether she took the emetics or not, and this statement exactly corresponds with the account given in other cases of urinous vomiting, in which, as in this case, the urine was vomited up, unmixed or not, according to the state of the stomach. It was indeed more likely that under these circumstances the urine would come up unmixed, the action of the emetic having expedited the expulsion of the morbid mass from her stomach.

Subsequent to April, when the urine had returned to its natural channel, and when it was reported to me the girl was considered an impostor, I paid personally particular attention to her case; and from the great misery I witnessed,

---

* A misunderstanding would very easily take place here, by the girl saying she vomited urine after taking an emetic.

I did

I did not doubt what I had not witnessed, both from her account, and from the analogy of similar cases from authors, living and dead, of the first celebrity. It was then that I prescribed some medicines for the girl, as detailed in the case, which were made up by the chemist, who, it is justice to observe, gratuitously supplied them, as well as those unprescribed by me, he had formerly sent from the time of my seeing her in December 1811. If this had not been the case, I would have procured them for her at the Infirmary, on account of the great misery and poverty of the object. Opium was her great consolation.*

As the catheter was never introduced, it was never ascertained by that means whether there was any collection of water in the bladder. More than once on visiting her, I past my hand over the lower part of the abdomen. I discovered no fullness such as a distended bladder would produce, but pain and soreness were complained of on pressure. For the heat and soreness of the private parts she applied some cooling ointment, which the mother procured from the druggist. In Dr. Senter's case, the urethra, bladder, and external genitals were so very sore, that it prevented him for some time, in addition to the dread she exprest of the instrument, from making an examination. The circumstance of soreness of the pudenda made me hesitate to get the instrument past, and on that ground I dropt an expression that the instrument would add to her sufferings. I felt a repugnance on my part to increase her pains for the gratification of my own curiosity, as I had in a great measure made up my mind to the nature of the case,—no fullness complained of, and none felt by manual examination; and as I did not think she would long survive, and the urinous vomiting had existed about five weeks before I saw her.† But if an examination had been made, the discovery of no urine, or of a quantity, in the bladder, would form no objection to a statement that it is vomited. Urinous vomiting can occur without any collection of urine in the bladder, which was the idea I had formed of the complaint, and the symptoms warranted the opinion. The absence, therefore, of pain and distension about the region of the bladder at the

* This drug was the great comfort in Dr. Senter's case; and it was necessary to have recourse to it in very large doses in the recent case with which Mr. Coley, of Bridgnorth, has favored the public, in the Medical Journal which has just come to hand, Dec. 3.

† If Mr. Gibbon had informed me of the resistance to the catheter, I certainly would have attended instantly, and I do not doubt the girl would have complied in my presence.

time time the suppression took place in October, is no ground for disbelieving the girl's story. No urine was collected in the bladder, at the time, to cause painful distension: considering also the highly morbid state in which she was, having twice vomited up her fæces during the first week of suppression; with this fæculent vomiting it is not unlikely some urine was thrown up. By referring to the case, it will be seen that two years before a suppression took place for two days and two nights, when, from the symptoms she describes, there appears to have been a collection of water in the bladder. Why in the first short suppression she should state the exact sensations, denoting the presence of water in the bladder, and in the second not, there can be no reason; but that she told the truth in both instances, and both accounts, are perfectly consistent with medical facts and experience.* Dr. Senter mentions that in his case the urine was vomited up for more than a week without the possibility of relief from the instrument, although it was sometimes kept in the bladder the whole night; clearly showing that although the vomiting of urine continued, none past from the bladder; and the authenticated circumstances in Dr. Girdlestone's communications, unequivocally prove the same thing. I subjoin these by permission of the doctor, with whom I have not the honor of a personal acquaintance, but who is well known as a literary character, and as a physician of accurate observation in extensive practice.†

---

*Yarmouth, Nov.* 22, 1812.

I have every reason to believe, from a case which came under my observation during the years 1796 and 1797, that a person may live many months without any secretion of urine by the kidneys. The young lady resided above fifty miles from this spot, and was the daughter of a respectable surgeon who had served in the medical staff in Germany. She had been under the care of her father and a physician for more than two years before she was sent to Yarmouth. Her brother having been drowned, had conspired, with other

---

* Upon this has Mr. Gibbon founded part of his charge of imposture. I speak to him medically, and upon the best authority, in saying, that in one instance it was the ischuria vesicalis where pain, distension, and great irritation to pass urine take place; in the other, the ischuria where no water is in the bladder, and where there is neither distension nor irritation to pass it. If he will look into Cullen and Sauvages, he will find I am correct.

† Dr. Girdlestone has also lately published an ingenious and critical pamphlet on the subject of the author of Junius's Letters.

events, to induce syncope, and a variety of nervous symptoms, which had resisted all the mineral, tonic, and nervous medicines that are commonly employed in such cases. When she reached Yarmouth, she had no use of her lower extremities, nor could she lie down without inducing hiccoughs or vomitings. She had two periodical fits of hiccough, which lasted for several hours out of the twenty-four. Either animal food or any liquid, immediately on entering the stomach, excited vomitings; and during the greatest part of her residence here she was obliged to live entirely on fruit, and to sleep in an erect posture in a chair. The liquid vomited up proved to be urinous, and on standing for some time, had the healthy nebula of common urine.

This young woman was about twenty years of age, fair, and not unhealthy in her complexion; and though she was rather full, yet she had more the appearance of a muscular than of a fat person. After having tried repeatedly pills with her, without the combination of any animal food in them, I had some beef pounded into pills, and covered over with the powder of gum tragacanth. Some of these pills were swallowed in the presence of Mr. Borratt, a surgeon of this town, and myself. We kept her in conversation for above twenty minutes, when she began to express her doubts of her being able to retain the pills, as she was feeling the same sensations as if she had animal food in her stomach. The pills, deprived of the greatest part of the gum, soon came up, and with them a considerable quantity of urine, which, on the admixture of quicklime, sent forth the ammoniacal smell. Under these symptoms she remained above two years, when the sudden death of her father obliged her to return to her mother; and in a few weeks after the poor girl died, without any inspection of the body having been permitted by the mother.

There were some facts which led me to believe that the stomach in this girl had really taken on, in some measure, the office of the kidneys. Her motions were invariably found to take on the shape of a distended rectum, and to have come away without a single drop of urine; and in the presence of myself and Mr. Borratt, the catheter was introduced by a late worthy practitioner, Mr. Downes, and the bladder was found not only empty, but so contracted as to lead us to believe that no urine had entered the bladder for some time. I have to add, that every medical man in this place at that time was made to bear witness to the case; and that the landlady, a most respectable woman, was sworn before a magistrate to the facts of the motions having always always come away without a drop of urine, and of her having seen the liquid repeatedly vomited up which had been proved to be urinous.

*June* 28, 1813.

The urine vomited up was about six or eight ounces at a time, about every six or eight hours, and it underwent the different appearances of the morning, midday, and evening urine, as in the urine which comes from the bladder of a person in perfect health, viz. if no animal food had been taken into the stomach to hasten the expulsion of the urine, which in that case only would be mixed with the food. And though I cannot be very accurate about the duration of this young lady's stay in Yarmouth, I think I may venture to assure you it was not less than two years from the time that I have great reason to believe she ceased to pass any urine by the bladder. I have read your ingenious paper on Ischuria, and besides the abundant evidence which you have collected from authors, I have read the evidence of others to prove the altered state of different glandular, secretions, but I think that your reasoning on glandular secretions cannot be controverted with effect without a new set of anatomical facts.* I have often seen the comatose symptoms of Ischuria suspended by urinary vomitings and perspirations.

*November* 14, 1813.

I have just received your letter, and I can have no objection to your making what use you please of my name on the subject of urinary vomitings. I am fully convinced of the existence of such cases, both by my own observations and those of medical writers. In my patient no emaciation or loss of the appearance of health took place during the whole of her residence in this place, which was till within a very few weeks of her death.

Mr. Gibbon has clearly described the meteorismus ventriculi of Sauvages.† Some years ago I had a lad who ceased to

---

* If Mr. Home prosecutes his ingenious experiments and inquiries, they may throw some light, much wanted, on this curious subject of vicarious secretions.

† Inflation of the stomach from internal disease. This is a curious circumstance. In the statement of imposture, a description is given of disease, which was immediately seen by this discerning physician, in reading the account in the Medical Journal; and this opinion was formed by Dr. Girdlestone and myself separately. Yet upon this has another part of the charge of imposture been founded. Mr. Short, surgeon to the Bedford Infirmary, and Mr. Leach, house-surgeon to the to discharge any urine by the bladder for three or four weeks at a time, during which interval he had daily urinary discharges by the bowels. He was in perfect health in every respect except this, and experiencing *an inflation* of the abdomen previous to the expulsion of his urine by the rectum. I doubted the case at first; but the urine coming away simply by the intestines was ascertained by the surgeon who had long attended the family, and witnessed the fact. The lad never was prevented from pursuing his trade, (which I think was that of a cooper;) and as it is many years ago, I am unable to say whether this occasional metastasis of the urine from the kidneys to the intestines ever entirely subsided.* I agree with you in thinking that the introduction of the catheter was an argument to crush all scepticism in my case, especially as the young lady had no previous knowledge of my intentions of having this examination made, which was immediately assented to; and the facts that are proved were that no urine was in the bladder, and that it was so contracted as to lead Mr. Downes and Mr. Borratt to conclude, that no urine had entered the bladder for some weeks. And on conversing with Mr. Borratt this morning, I find that at different intervals he had repeated the introduction of the catheter in this patient, without finding any alteration in the state of the bladder, or any urine in it.

---

In addition to these decisive communications from Dr. Girdlestone, I have been favored with a communication by an ingenious physician in Dublin, mentioning that in a patient in whom urinous vomiting had occurred, that species of vomiting had ceased many months before the death of the patient.

That an opinion may be formed how far I was or was not justified in believing that vomiting of urine was the ground on which the girl was objected to, I here add a copy of the document drawn up at the meeting of the medical gentlemen, and which was delivered to me by Mr. Gibbon.

"We, the undersigned, having this day collectively examined and made inquiry into the case of Ann Fooks, and

---

the same, (the last gentleman at my request saw the girl during the suppression,) are of opinion, *from their own examination,* that the tumefaction over the region of the stomach, was a distension of that organ from disease. There could be no doubt about its nature.

* This case remarkably confirms those of Baglivi, Haller, Hildanus, and Morgagni. See paper on Ischuria, in Medical Journal for May, p. 361.

having having heard her positive assertion that she did not void a drop of urine from the bladder for the space of twenty-six weeks and two days,* are unanimously of opinion that such an assertion is totally void of all probability of truth; and we are likewise of opinion, that her statement of her urine having been thrown up by the mouth during the whole course of that time, is equally void of all probability, and appears to us utterly impossible.

"*Bedford, June* 26, 1812."

What is the natural inference from reading this document? That the girl was objected to on account of the belief of the total improbability of her assertion that she past no urine from her bladder for the time specified, and of the utter impossibility of her having vomited it up during the same period: and as vomiting of urine, and nothing but vomiting of urine, was the original matter of dispute, to which no doubt its occurrence in the case gave rise, what other conclusion could I in particular draw from the paper in which no other circumstances but the suppression and vomiting of urine are stated?† Now I did dissent, and I beg leave still to dissent, from this declaration of utter impossibility, and I do so on the authorities of professional men of the first respectability, and of the best records of ancient and modern date.‡ Would the girl's case have excited attention but for the vomiting of urine? I most certainly had my doubts at first, but a consideration of the peculiarities of the case, reading, correspondence with men of eminence and research, gradually removed my doubts, and I ended

---

* This is an accidental error of no consequence; it was twenty-six weeks all but two days. This document I have inserted since the appearance of Mr. Pulley's paper, who has given the names of the gentlemen who signed it; it was therefore right that the document itself should appear.

† Vomiting of urine was the chief feature which occupied the conversation when Mr. Short, Mr. Pulley, Mr. Gibbon, and myself, met at Ann Fooks's, who was severely questioned by Mr. Gibbon on the subject, but who repeatedly asserted that she vomited it. This was the head and front of her offending. We then separated with our different opinions on the subject. What discussion subsequently took place at the meeting with the other professional gentlemen, at which I was not present, and at which the document was drawn up in one united opinion, I do not know. I beg it may be understood, that I do not intend to say that other circumstances of the case were not taken into consideration at this meeting; all I mean to say is, that vomiting of urine was the original objection to the girl by Mr. Gibbon, and was the cause of the *original* difference of opinion between us.

‡ Haller, Morgagni, Sauvages, Girdlestone, &c. &c.

in the belief that the circumstances stated in the document are by no means utterly impossible, particularly too in a question of intricacy.* Let the case and all the circumstances connected with the girl's statements be dispassionately examined, let the opinions and authenticated facts of discerning characters of the present day be canvassed with respect, and let the great medical authorities be looked into with candor, and then let judgment be given.

The objections to the girl's statement are not to me sufficient grounds for rejecting her. The circumstance of the urine being thrown up unmixed is certainly very probable, for the reasons already given, and from the close and undeniable analogy which it bears to the same facts in the cases of Dr. Girdlestone and Dr. Senter. In addition, Mr. Coley states, in his recently published case, that the vomited urine, *minutely examined*, possessed all the properties of healthy urine. The resistance to the use of the catheter can be readily accounted for from what has been mentioned, in addition to which it is clear that no water was collected in the bladder to cause distension and pain sufficient to overcome her feelings against the use of it; the argument, therefore, that the great pain of suppression would have been sufficient to have induced her to comply, falls to the ground, although the suppression should have existed for a considerable time.

Her extreme suffering from disease in various shapes, appears to me to preclude the idea of her inventing so extraordinary a circumstance as urinous vomiting. There are abundant sources for commiseration without it, and if invented would have been easily detected, did not her other symptoms materially corroborate the fact. Again: had she been in a better state of health than that in which she really was, this would not preclude the possibility, medically speaking, of vomiting of urine, from the analogy of the facts in Dr. Girdlestone's cases, where no emaciation was observable.† What symptoms were wanting to complete the species of stupor which occurred immediately previous to the return of the urine to its natural passage, I cannot

* Surely it cannot be imagined I mean the slightest disrespect to any individual by this dissent, as it is only that difference of opinion which we daily meet with. The question then is now, whether the circumstances stated in the document are sufficiently improbable and impossible to convict the girl of imposture.

† See Mr. Coley's case, which confirms Dr. Girdlestone's. "The pulse continued at 90, the appetite was good, and *she had not experienced any emaciation*," are Mr. Coley's observations on his case of urinous vomiting.

divine, divine. The account of this comatose state was taken from the lips of rustic ignorance, and is the description of exquisite disease, constituting the carus ischuriosus, or delirious stupor from suppression of urine. I think it very probable if the brain had not been relieved, the girl would have died of oppressed brain, and a urinous fluid would have been found effused into its ventricles.* Whether then the objections to the girl are founded on her statement of the duration of the suppression of urine, of the vomiting of it at intervals, for the time specified, of the different symptoms attending the commencement of the suppression at two separate times, on the distension of the abdomen, on the appearance of the stupor, on what is considered her more healthy appearance,† or on all of these, I cannot join in the cry against her, because the very same things have been witnessed in the patients of very respectable professional men of discernment and accuracy of the present day, with many others, which the paper on Ischuria will furnish; and having occurred in them, I cannot see why the possibility of such occurrences should be denied in the unfortunate sufferer, particularly under her highly morbid condition.

The greatest part of her sufferings were in a tangible shape, and were frequently witnessed by some of the most respectable people in Bedford. They were visible in the shape of palsy, spasms of the limbs, vomiting of blood, discharges from the ears, profuse sweatings, cutaneous eruptions, and dropsical swellings. They were not the transient effects of ephemeral disease, they made a permanent impression for a considerable time. The parish relief was at one time withdrawn, when it was renewed by the order of an active magistrate, not easily deceived, who has frequently personally examined and pitied her sufferings and her indigence. A great part of the observations on the case were written a considerable time ago, and are now interwoven with other matters which Mr. Gibbon's paper has rendered it necessary to detail. Had I done any thing beyond defending an opinion on urinous vomiting, which I still maintain, and which it was necessary in justification to defend, a

---

* See Cullen and Sauvages for Carus and Coma; Portal and Cheyne, in the paper on Ischuria, p. 368, the latter on Apoplexy passim; and p. 212, where a recovery took place from this urinous stupor, by the urine unexpectedly returning to its natural channel.

† The expression "very thin," used by Mr. Pulley, is analogous to emaciation. It is a relative term applied by me to the different states in which the girl was before and after the stomach complaint commenced.

retort retort of dignified resentment and of calm refutation would have given grace to his opposition, and a professional spirit to his paper. It must now be recollected, that whatever remarks have come from me, have been called forth in *defensive reply*. Unfortunately, however, for his cause, the disputed occurrence has been confirmed as an unequivocal fact by Haller, Morgagni, Sauvages, Girdlestone, and a host of medical writers, whose authorities have never been doubted, and whose publications are considered at this day as standard books of reference.

The girl has sworn that she past no urine naturally for the space of time mentioned in the case, and that during that period she very frequently vomited a fluid of the taste, color, and smell of urine. Two other persons have sworn, one that she saw her vomit a fluid similar to that which was brought to me, and that she was in the room some time, and should have seen if she had drank any thing previous to vomiting.* The other, that she saw her vomit a dark slimy matter of a nauseous smell like strong urine.† I put these persons to their oaths with a view of seeing whether they would bear the test; the oaths were taken with great readiness, and with every appearance of truth. I never had any idea of treating the subject but purely as a medical question, and as such I have discussed it both now and in a former paper. The judicial mode of inquiry has been forced upon me, and in addition to the oaths I subjoin the testimony of three most respectable members of society to the girl's character, that it may be seen I have not taken up the history on light grounds, whether medically or judicially considered. This paper was sent to me without any knowledge of it whatsoever on my part until the moment of its reception, which was on the 29th of March, 1813. The document itself has no date, and as far as I am acquainted, I do not believe that the girl knows to this day that I possess such a testimony to her character.

"Whereas the character of Ann Fooks, a poor young

---

* Sarah Triplow, æt. 44, in whose cottage the girl lodges. This woman says she has witnessed her vomiting urine more than once.

Hannah Davies, another woman, also says that she saw Ann Fooks vomit a fluid similar in color and smell to urine, and that she was in the room some time before. This woman does not live in the same house. She is ready to depose to this declaration.

† Lætitia Clarke, æt. 67, who lives in a row of cottages at a small distance.—The oaths were administered by Francis Green, esq. a justice of the peace, before the Rev. Thomas Cave, the clergyman of the parish in which the girl resides, and the Rev. T. S. Grimshaw, vicar of Biddenham, and myself, in July, 1812.

woman, woman, who has for several years resided in Bedford, under a succession of most painful and distressing bodily complaints, has, we apprehend, received injury from various imputations tending to lessen that esteem and respect to which we think her entitled; we, therefore, whose names are hereunto subscribed, not presuming to offer any opinion on points exclusively of a medical nature, but actuated by compassion for her long sufferings, and a desire of vindicating her integrity and veracity, do hereby think it right to state that we have had frequent opportunities of estimating her moral character, in the course of very frequent visits during her long protracted illness, in consequence of which, altogether unsolicited by herself, we do voluntarily state our opinion, that whatever may be the peculiarities of her case, medically considered, we deem her by no means justly liable to the charge of wilful deceit and imposture: on the contrary, we think she has evinced the most decided proofs of piety and resignation under afflictions the most extraordinary and painful.

(Signed) "LEGH RICHMOND, Rector of Turvey.
"T. S. GRIMSHAW, Vicar of Biddenham.
"J. K. MARTYN, Curate of Pertenhall."

Certainly, Mr. Pulley did state to me an opinion of urinous vomiting similar to that which he has described in his published paper. I never heard the doctrine maintained that solid calculous matter deposited in the bladder was absorbed, carried to the stomach, and vomited as such; but as vomiting of urine is a fact established upon unquestionable authority, I see no reason why a calculous precipitation should not take place from the urine in the stomach as well as in the bladder. In Mr. Coley's case a disagreeable excess of uric acid attended the vomiting of urine; this in a sabulous form would be calculous matter. What says Haller?—Arenosæ enim glebulæ naribus adhæserunt. What is the saline deposition on the skin in urinary perspirations but the same thing?* What are urinous particles, solid or fluid? Are they muriatic or phosphoric salts, lithic acid, phosphat of lime, animal extractive matter, or all these put together? The component parts of urine in solution is urine, nothing else can be made of it. The discovery of no stone in the dissection of Dr. Senter's patient, forms no objection to the case in the least; one may have been in the bladder at the examination, and yet none found at the death of the patient; for there is a species of stone friable and easily crumbled

* See p. 359 of the paper on Ischuria, for a case of this kind, by Dr. Johnson, of Kidderminster.

 down down into small portions and past off.* Calculi of this kind are sometimes formed, in which the uric acid is not deposited in crystals, but is mechanically mixed with the animal mucus of the urine. The particular kind of gravel which came away from Lucy Foster, would produce a stone of this kind. What says Dr. Senter? "A *brick-colored* gravel began to pass off through the catheter, and soon became so large and plentiful, that neither urine or gravel could be completely evacuated by the instrument in the usual form." Calculi (Phil. Trans. 1808) of uric acid vary in color from a *deep reddish brown* to a pale yellowish brown; the former was the kind of calculi which came away from Foster, and would produce a stone exactly like the one Dr. Senter says he felt with the sound. "I readily met with a stone which appeared of a small size, and rather softer than urinary calculi usually are."

If it be admitted that Foster drank her urine for three long years, to which I cannot agree, so diseased as she was, I do not see how it was to be vomited unmixed with the contents of the stomach, taken in this way, any more than when it is past into the stomach by the animal economy; and that it was brought up like healthy urine in this case as well as in Dr. Girdlestone's and Mr. Coley's cases, there is given the most undeniable testimony. Under the same supposition, how was it that the urine past off by the navel? as also happened to the young woman mentioned by Hildanus. No fluid was discovered after death with a urinous impregnation, because it was perfectly plain that the urine was all discharged by vomiting and purging before death; and if any small quantity did remain in the stomach, its detection would be extremely difficult, from the gangrenous state of that viscus, and the foetid nature of its contents. The editors of the Medical Facts, at the head of whom was the late celebrated Dr. Samuel Foart Simmons,† have abridged into their works this case of Dr. Senter's, and have said that Dr. Senter has added many judicious remarks. The genius of Darwin has quoted this case as one of authenticity, in confirmation of some of his physiological opinions.

The medical records to which I originally referred for a

* See Wollaston, Home, and Brande, in Phil. Trans. on urinary calculi. Dr. Wollaston describes six kinds. Surgeons have often seen quantities of sabulous particles, and small fragments, (not distinct ones themselves,) of evidently larger stones discharged by the urethra.

† This learned gentleman was physician to St. Luke's for many years, and was called upon to attend the King.

proof proof of the frequent occurrence of the disputed symptoms, are those of authors whom the medical student is taught to venerate and to respect; to look up to as containing a fund of knowledge and experience to be drawn upon by the profession. These medical patriarchs are dead, but as long as their works live they will never be dead to society. They have niches in the temple of learning which nothing but oblivion can destroy. They are not to be blown away by the blowing of the breath of displeasure, like dust upon the shelf; but will remain a ponderous mass of accumulated wisdom, which something more than individual disregard can erase. The facts will remain, but medical systems will change—they must change, because human knowledge in the application of these facts is progressive: but amidst these revolutions, honor will continue to be paid to the memory of such as these, for their accuracy and professional zeal, as long as learning and genius are esteemed among mankind.

---

*January* 4, 1814.

P. S. I have just read Mr. Gibbon's paper. Mr. Gibbon need not have gone so far back as the Journal for July for my belief that the meeting of the medical gentlemen, which took place when the document was drawn up, was a casual one. I have stated this also in my paper to which Mr. Gibbon has just replied, and I again repeat that I have no doubt the meeting was perfectly casual and accidental. It was stated that all the gentlemen of the faculty who had attended the patient should be present at the proposed investigation, and that it was proper I should be there. This was the hint I alluded to, and Mr. Gibbon expressed his sorrow at my absence; yet he holds the meeting, when he learnt by sending to my house that I was from home, and could not be in the way in time to attend; and afterwards publishes a statement in which he tells the public I am implicated, and in the full and final discussion of which I had no part, as all the medical gentlemen at this meeting had never met together before on the subject; and Mr. Gibbon should have recollected that between him and me the original difference of opinion took place on the case. This certainly wears an ungracious appearance on his part. All the other gentlemen who were called to attend this meeting, did, and could do no otherwise than attend and declare their opinions on the symptoms of the disputed case, and this was done in a fair and manly manner. I never intended the smallest idea of unfairness against them. It was Mr. Gibbon's mode of proceeding relative to this business, subsequent to the meeting as already stated, which created created an ungracious feeling in my mind on that account, and not any thing done by the gentlemen in attending this meeting; and my observations apply this way in my former paper. I do not forget the other meetings prior to this I was present at, but neither Dr. Kerr nor Dr. Alvey were present. What therefore Mr. Gibbon means when he says that I was present at a meeting of *all* those medical gentlemen to whom I allude, I do not understand. The ludicrous history was or was not intended to apply to the cases related on the great authorities stated in the paper on Ischuria. If it was intended, the observations which I in consequence made on the disregard of learned authorities, still hold their ground; if it was not intended as the medium of conveying a sarcastic disbelief of the statements taken from those high professional characters, to whom Mr. Gibbon now offers the homage of respect, then the cases they relate are cases of credit, and being fully as extraordinary as the symptoms stated in the case of Ann Fooks, the opinion of the impossibility of their occurrence in her falls to the ground on the best authorities. This was exactly the Q. E. D. of that paper.

*Bedford.*

---

*For the Medical and Physical Journal.*

